# Serial troponin measurements to monitor risk and response to endothelin A antagonism in chronic kidney disease

**DOI:** 10.1093/ndt/gfaa214

**Published:** 2020-09-27

**Authors:** Atul Anand, Tariq E Farrah, Eve Miller-Hodges, Anoop S V Shah, Fiona E Strachan, Edwin Carter, Neil R Johnston, David J Webb, Nicholas L Mills, Neeraj Dhaun

**Affiliations:** 1 University/British Heart Foundation Centre of Research Excellence, Queen's Medical Research Institute, University of Edinburgh, Edinburgh, UK; 2 Department of Renal Medicine, Royal Infirmary of Edinburgh, Edinburgh, UK

Chronic kidney disease (CKD) is a global epidemic, but efforts to reduce its burden have been less effective than for other non-communicable diseases. Between 1990 and 2017, the global age-standardized mortality rate fell by 41.3% for chronic obstructive pulmonary disease, 30.4% for cardiovascular disease, 14.9% for cancer, but by only 2.8% for CKD [1]. Importantly, CKD is an independent risk factor for cardiovascular disease (CVD), which is its most common endpoint [2]. Indeed, in a recent analysis, almost 7% of global CVD burden was attributed to impaired kidney function [3]. Identifying cardiovascular risk early in CKD and assessing its response to treatments are key unmet clinical challenges.

Circulating cardiac troponin is a strong independent predictor of cardiovascular morbidity and mortality in individuals with and without CVD [4]. We recently demonstrated that cardiac troponin concentrations are reduced by statin therapy and, importantly, the magnitude of this reduction is an independent predictor of future coronary events [5]. This was the first description of a biomarker that can dynamically track cardiovascular risk with time and raises the possibility that serial testing may have a role in assessing response to a range of therapies. Given increased troponin concentrations are associated with higher blood pressure (BP) [6], a common feature of CKD [7], we hypothesized that in those with CKD, troponin concentrations may also be modified by BP-lowering therapies, a cornerstone of CKD management. To explore this, we performed a *post hoc* analysis of a previously reported randomized, double-blind, placebo-controlled study that examined the systemic cardiovascular and renal effects of a selective endothelin A (ET_A_) receptor antagonist [8]. Endothelin receptor antagonism is a potential novel therapy for CKD currently being studied for its BP-, lipid- and proteinuria-lowering effects [9] as well as its ability to improve patient outcomes [10].

In a randomized, double-blind, three-way crossover study, 27 participants with optimally managed and stable non-diabetic proteinuric CKD received the selective ET_A_ receptor antagonist sitaxentan, nifedipine once daily or a matched placebo for 6 weeks in addition to their usual medications. There was a minimum 2-week washout period between phases. All subjects completed all three phases of the study. Patient diagnoses were immunoglobulin-A (IgA) nephropathy (*n* = 14), focal and segmental glomerulosclerosis (*n* = 6), membranous nephropathy (*n* = 3), hypertensive nephrosclerosis (*n* = 2), reflux nephropathy, microhematuria of presumed glomerular origin (*n* = 1 for both) and one subject with an unknown cause for their CKD. The mean age was 48 ± 12 years, mean body mass index was 29.3 ± 4.6 kg/m^2^ and 23 participants were male. The mean estimated glomerular filtration rate was 54 ± 26 mL/min/1.73 m^2^ and mean proteinuria was 2.0 ± 1.7 g/day. Eighteen (67%) participants were on statins and 24 (89%) were on either an angiotensin converting enzyme inhibitor or an angiotensin receptor blocker.

High-sensitivity cardiac troponin I concentrations (Abbott Laboratories, Abbott Park, IL, USA) were measured in stored plasma at baseline and at 3 and 6 weeks during each phase (limit of detection 1.2 ng/L, interassay coefficient of variation (CV) <10% at 4.7 ng/L) [11]. Low-density lipoprotein (LDL) cholesterol was measured by a direct method using an enzymatic colorimetric assay; the limit of detection was 1.0 mg/dL with an intra- and interassay CV of 1.4% and 2.2%, respectively. Approval for the study was granted by the local ethics review board and participants provided informed consent. The study was performed in keeping with the principles outlined in the Declaration of Helsinki.

At baseline, the median troponin concentration was 2.3 ng/L (interquartile range 1.6–4.4), the mean systolic BP was 125 ± 12 mmHg and the LDL cholesterol concentration was 103 ± 32 mg/dL. There were no differences in any of these parameters at the start of each phase (P > 0.05 for all). Whereas placebo and nifedipine did not affect cardiac troponin, selective ET_A_ antagonism reduced troponin concentrations by 1.6 ± 0.5 ng/L (27 ± 19%) (P = 0.01 versus both placebo and nifedipine; Figure 1A). LDL cholesterol was unaffected by placebo or nifedipine, but ET_A_ antagonism decreased this by 19 ± 4 mg/dL (20 ± 13%) (P < 0.001 versus both) [12].

**FIGURE 1:**
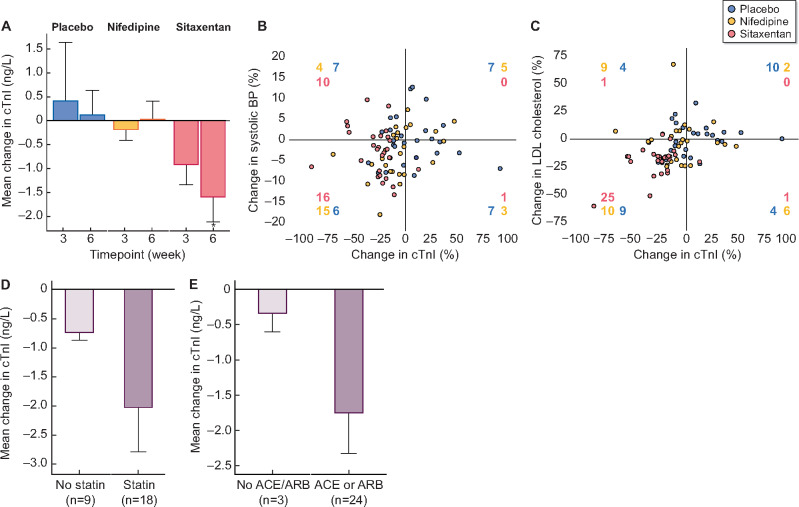
(**A**) Absolute change ± standard error (SE) in cardiac troponin I after 3 and 6 weeks of treatment with placebo (blue), nifedipine (green) and sitaxentan (red). (**B**) Percentage change in systolic BP and (**C**) LDL cholesterol plotted against the percent change in cardiac troponin across the three treatment arms. The numbers in each quadrant in (B) and (C) represent the number of patients (from a total of 27) that achieved the relevant changes in the parameters shown by treatment. For example, 25 patients demonstrated a reduction in both LDL cholesterol and troponin I compared to 9 with placebo and 10 with nifedipine (C). (**D**) Absolute change ± SE in cardiac troponin I after 6 weeks of treatment with sitaxentan in patients prescribed (dark blue) and not prescribed (light blue) a statin at baseline. (**E**) Absolute change ± SE in cardiac troponin I after 6 weeks of treatment with sitaxentan in patients prescribed ( dark blue) and not prescribed (light blue) a renin–angiotensin system blocker at baseline. *P < 0.01 versus placebo and nifedipine. Baseline cardiac troponin and LDL cholesterol levels were assessed by one-way analysis of variance (ANOVA) for a period effect at the start of each treatment phase. Changes in troponin and LDL concentrations from baseline were assessed using paired *t*-tests with Week 6 data. Changes in troponin and LDL concentrations between phases were assessed using two-way ANOVA with Tukey’s multiple comparison test. Data were analysed in R (version 3.3.3; R Foundation, Vienna, Austria). Significance was taken at the 5% level.

While individual changes in systolic BP varied across each study phase and were unrelated to changes in troponin concentration (Figure 1B), decreases in LDL cholesterol were associated with reductions in troponin concentration on ET_A_ antagonism (Figure 1C). Interestingly, the troponin-lowering effects of selective ET_A_ receptor antagonism tended to be greater in those already taking a statin (–2.0 ± 0.8 versus –0.7 ± 0.1 ng/L without statin, P = 0.11; Figure 1D) as well as in those patients prescribed a renin–angiotensin system blocker at study entry (–1.8 ± 0.6 versus –0.3 ± 0.3 ng/L without treatment, P = 0.04; Figure 1E).

Our observations suggest that troponin concentrations are not reduced by BP-lowering therapies in CKD, at least not within 6 weeks of initiating treatment. However, the LDL cholesterol–lowering effect of ET_A_ antagonism was associated with a rapid and reproducible reduction in troponin concentration. These findings are consistent with our previous observations [5] and suggest that LDL cholesterol may be an important determinant of troponin concentration. While the mechanism through which selective ET_A_ receptor antagonism reduces LDL cholesterol is unknown, our findings are novel and suggest that ET_A_ receptor antagonism has the potential to improve cardiovascular health in those with optimally managed CKD by reducing BP, lowering LDL cholesterol and reducing myocardial injury.

The recent Study of Diabetic Nephropathy with Atrasentan (SONAR) study demonstrated the efficacy of atrasentan, an ET_A_ receptor antagonist, in slowing diabetic CKD progression [10]. Interestingly, the benefits of atrasentan on CKD progression were seen regardless of proteinuria reduction, suggesting additional mechanisms. Also, in this same study, atrasentan reduced the incidence of non-fatal stroke. Our current data suggest that these beneficial effects of an ET_A_ blocking approach may relate to reductions in circulating LDL cholesterol and cardiac troponin, although studies with longer follow-up to accrue cardiovascular events are now required. ET_A_ receptor antagonism offers a potential novel strategy to reduce cardiovascular risk in patients with CKD and further investigation of its mechanistic links with LDL cholesterol and cardiac troponin are merited. A Phase 2 study of selective ET_A_ antagonism in combination with angiotensin II receptor blockade in patients with proteinuric CKD is ongoing [13] and should help confirm the current observations.

In this proof-of-concept study, improvements in these risk factors were achieved despite high baseline use of statins and renin–angiotensin system blockers. Further studies are required to determine whether prolonged reductions in BP can alter left ventricular mass and reduce cardiac troponin concentrations and to establish the role of serial troponin testing in monitoring the response to novel and established cardiovascular therapies in those with and without CKD.

## FUNDING

This work was supported by the British Heart Foundation (Butler Senior Clinical Research Fellowship FS/16/14/32023 to N.L.M., Intermediate Clinical Research Fellowship FS/13/30/29994 to N.D.).

## CONFLICT OF INTEREST STATEMENT

A.A.S.V. has received honoraria from Abbott Diagnostics. D.J.W. has acted as a consultant for Idorsia. N.L.M. has acted as a consultant for Abbott Diagnostics, Roche and Singulex. N.D. has acted as a consultant for Retrophin. All other authors have no financial disclosures. The sponsors or funders had no role in the design and conduct of the study; in the collection, analysis and interpretation of the data; or in the preparation, review or approval of the manuscript. The results presented in this article have not been published previously in whole or part.
